# Extracellular Vesicles as Protagonists of Diabetic Cardiovascular Pathology

**DOI:** 10.3389/fcvm.2017.00071

**Published:** 2017-11-09

**Authors:** Dakota Gustafson, Shawn Veitch, Jason E. Fish

**Affiliations:** ^1^Department of Laboratory Medicine and Pathobiology, University of Toronto, Toronto, ON, Canada; ^2^Toronto General Hospital Research Institute, University Health Network, Toronto, ON, Canada; ^3^Heart & Stroke Richard Lewar Center of Excellence in Cardiovascular Research, University of Toronto, Toronto, ON, Canada

**Keywords:** extracellular vesicles, diabetes, cardiovascular, atherosclerosis, cardiomyopathy, miRNAs

## Abstract

Extracellular vesicles (EVs) represent an emerging mechanism of cell–cell communication in the cardiovascular system. Recent data suggest that EVs are produced and taken up by multiple cardiovascular cell types, influencing target cells through signaling or transfer of cargo (including proteins, lipids, messenger RNA, and non-coding RNA). The concentration and contents of circulating EVs are altered in several diseases and represent explicit signatures of cellular activation, making them of particular interest as circulating biomarkers. EVs also actively contribute to the progression of various cardiovascular diseases, including diabetes-related vascular disease. Understanding the relationships between circulating EVs, diabetes, and cardiovascular disease is of importance as diabetic patients are at elevated risk for developing several debilitating cardiovascular pathologies, including diabetic cardiomyopathy (DCM), a disease that remains an enigma at the molecular level. Enhancing and exploiting our understanding of EV biology could facilitate the development of effective non-invasive diagnostics, prognostics, and therapeutics. This review will focus on EV biology in diabetic cardiovascular diseases, including atherosclerosis and DCM. We will review EV biogenesis and functional properties, as well as provide insight into their emerging role in cell–cell communication. Finally, we will address the utility of EVs as clinical biomarkers and outline their impact as a biomedical tool in the development of therapeutics.

## Introduction

The prevalence of diabetes mellitus (DM), especially type 2 DM (T2DM), is steadily increasing and is predicted to rise substantially over the next decade (1, 2). Mortality rates of individuals with T2DM are consistently elevated, with an overall excess risk of death from any cause of ~27% (3). There is abundant epidemiological and mechanistic evidence underscoring the role of T2DM as an independent risk factor for accelerated cardiovascular disease (4, 5). Individuals with T2DM are at high risk for developing several cardiovascular disorders, including coronary heart disease, stroke, peripheral arterial disease, and diabetic cardiomyopathy (DCM) (6). Much of the vascular burden associated with T2DM is caused by the chronic, injurious effects of hyperglycemia on the micro- and macro-vasculature [see Ref. (7) for a comprehensive review]. Indeed, many of the earliest pathological responses to hyperglycemia are manifested in the vascular endothelial cells (ECs) that interface with elevated blood glucose levels. Traditionally, the activation of pathological inflammatory processes through both paracrine and endocrine cellular communication has served as the centerpiece for the purported development of diabetic cardiovascular pathologies (8). However, a third mechanism of intercellular communication, involving the intercellular transfer of extracellular vesicles (EVs), is emerging as an important mediator. Much remains to be explored regarding the contribution of these EV pathways to cardiovascular complications in T2DM patients.

## Extracellular Vesicles

### Nomenclature and Biogenesis

**Figure 1:**
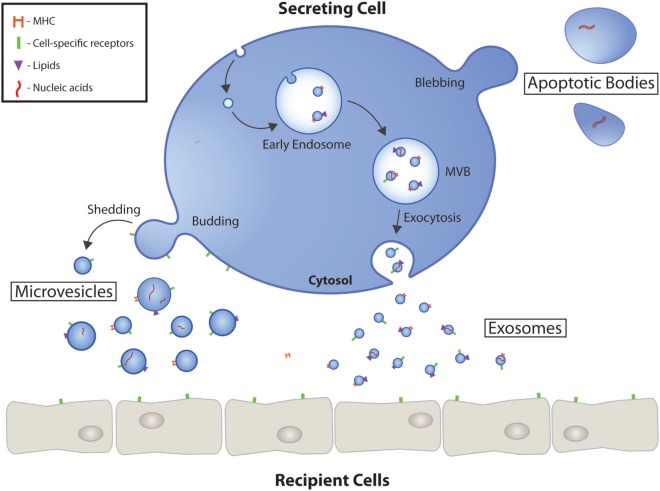
Extracellular vesicle (EV) biogenesis and secretion. Schematic representation of the origin and release of EVs by eukaryotic cells. Exosomes are formed as intraluminal vesicles by budding into early endosomes. MVBs typically have two fates; fusion with lysosomes or fusion with the plasma membrane, which allows the release of their content into the extracellular milieu. Microvesicles arise as a result of outward budding and fission of the plasma membrane mediated by phospholipid redistribution and cytoskeletal protein contraction. The largest EVs, apoptotic bodies, are formed during programmed cell death mediated in part by actin-myosin mediated membrane blebbing. EVs have numerous markers ranging from proteins, to lipids, to nucleic acids. MVB, multivesicular body.

Extracellular vesicles are a heterogeneous population of small cell-secreted phospholipid bilayer-bound structures naturally released into the extracellular space. Secretion of EVs appears to be conserved across species, as they have been identified in fundamentally all eukaryotes and many prokaryotes (9). Using current conventions, EVs are classified into three major subtypes based on biogenic, morphological, and biochemical properties: exosomes, microvesicles (MVs), and apoptotic bodies (Table 1). Characterization and classification of this heterogeneous population of membrane vesicles has been challenging and the source of heated debate, but based on current evidence, a working basis for a consensus has recently been reached (10). Garnering focused attention have been exosomes, which are the smallest subgroup of EVs at approximately 30–100 nm in diameter. Exosomes are generated within the endosomal system, initially forming as intraluminal vesicles inside multivesicular bodies (MVBs) in the endosomal compartment during the maturation of early into late endosomes (Figure 1) (11). The formation of MVBs has been shown to be mediated by the endosomal sorting complex required for transport (ESCRT) machinery, which sequesters ubiquitinated transmembrane proteins and drives intraluminal membrane budding (12, 13). However, ESCRT-independent exosome biogenesis pathways have been suggested, primarily *via* tetraspanin-dependent mechanisms (14, 15). MVBs have a bipartite fate; either degradation through fusion with lysosomes or exocytosis as exosomes after fusion with the plasma membrane. The release of exosomes into the extracellular milieu appears to be facilitated, in part, by SNARES and Rab proteins (16).

**Table 1:** Common classifications of extracellular vesicles (EVs).

Characteristics	EVs		
	Exosomes	Microvesicles	Apoptotic bodies
Biogenesis	Sorted as intraluminal vesicles in multivesicular endosomes and secreted after the fusion of multivesicular bodies with the plasma membrane	Fission and outward budding from the plasma membrane directly into the extracellular environment	Generated through apoptotic fragmentation and blebbing
Size	30–100 nm	100–1,000 nm	1–5 µm
Markers	Tetraspanins (CD9, CD63, CD81), heat shock proteins (HSPA8, HSP70, HSP90), Annexin A2, Enolase 1, Flotilin-1, and TSG101		TSP, 3Cb
Cargo	DNA, RNA (messenger RNA, miRNA, lncRNA), Proteins (cytokines), Lipids		

Microvesicles (also known as microparticles or ectosomes) tend to be larger in size, approximately 100–1,000 nm in diameter, and arise in a biogenically distinct fashion. They are formed by the outward budding and scission of extracellular membrane (Figure 1) (17). The release of vesicles is preceded by the budding of cytoplasmic protrusions, which detach through the fission of their stalk. It is thought that dynamic interactions between cholesterol-rich microdomains regulated by animophospholipid translocases initiates formation, followed closely by vesicle budding induced by translocation of phosphatidylserine to the outer-membrane leaflet and contraction of cytoskeletal structures by actin-myosin interactions (18, 19).

Apoptotic bodies are the largest subtype of EVs, encompassing a wide size range of approximately 1–5 µm in diameter. Unlike exosomes and MVs, which are generated in both physiological and pathological conditions, apoptotic bodies are only generated by plasma membrane blebbing of apoptotic cells (20). While commonly regarded as purely cellular debris, in the emerging context of EV cellular communication, apoptotic bodies represent a potentially untapped source of biologically useful information; having various cargoes, including organelles, packed tightly within their structures (21).

### Function

Extracellular vesicles are secreted from most cell types and are able to elicit diverse responses in recipient cell types. This is accomplished by engagement of EV surface proteins with receptors on recipient cells or through internalization of EVs into recipient cells, thereby transporting EV cargo into the cell. Uptake mechanisms include endocytosis, fusion with the recipient cell’s membrane or uptake *via* binding of EV surface proteins such as tetraspanins to the target cell’s membrane (22, 23). The notion that exosomes and MVs act as effectors of cellular communication is founded on ample data showing that they can transport bioactive molecules to target cells—either locally, or systemically by entering biological fluids—and transfer select cargo to affect molecular pathways and the behavior of recipient cells (24–26). Cargo can include genetic material such as DNA, messenger RNA, non-coding RNA (e.g., miRNA), as well as proteins, carbohydrates, lipids, and in unique circumstances, organelles such as mitochondria. Regarding cell–cell communication, while many avenues of effector action have been described, EV-associated miRNAs have received the most thorough examination. They serve as potent biomolecules that direct multiple cellular processes *via* negative regulation of target genes at the posttranscriptional level (27). Distinctive surface markers including cellular receptors and transmembrane proteins on both exosomes and MPs appear to provide a means of increasing cellular interaction specificity. *In vitro* findings have also shown distinct cargo, including genetic material, proteins, and other molecules, in exosomes and MPs, and correspondingly discrete functions (28, 29). Finally, although less well studied than exosomes and MPs, apoptotic bodies have been suggested to harbor functional capabilities, in particular, carrying miRNAs known to direct vascular protection (30).

### EV Enrichment

Although there is an intense focus on the biogenesis, cargo, and subsequent function of EVs and their heterogeneous subpopulations, many efforts are stifled by limitations imposed by current isolation and characterization methodologies. There are many strategies available for the enrichment of EVs, the most popular being ultracentrifugation, size exclusion chromatography, and commercially available EV precipitation kits. Ultracentrifugation is the gold standard for EV isolation, being used in more than 50% of reports (31, 32). Differential ultracentrifugation employs a series of centrifugation cycles with varying centrifugal force and duration, escalating from 400 to 100,000 *g*, leading to the preferential isolation of EV subtypes that have unique densities; apoptotic bodies (2,000 *g*), MVs (10,000–20,000 *g*), and exosomes (≥100,000 *g*) (33–36). Although standard ultracentrifugation offers a relatively pure sample, it can also precipitate large proteins not associated with EVs. High centrifugal forces can also be potentially damaging to EVs. These protocols are also particularly time intensive, require expensive equipment, and are difficult to implement with small amounts of starting material (33). More recently, contamination by protein and particle aggregates has been partially addressed through the adoption of density gradient centrifugation, which utilizes density gradients to separate specific EV populations (32).

Other techniques have been developed to better meet time, sample quantity, and equipment sensitive situations. In particular, many commercial kits offer comparatively rapid precipitation of EVs through the incorporation of polymers such as polyethylene glycol (37). However, preparations from commercial kits have been shown to have low purity and potentially impaired functionally due to co-precipitation of non-vesicular contaminants such as lipoproteins and polymer material (38). As a result, commercial kits may be more suited for high-throughput EV cargo characterization, such as miRNA profiling. Size-based EV isolation techniques, such as filtration and size exclusion chromatography, are also available (39, 40). Such methodologies offer several advantages, including moderately rapid isolation, ease of use, reduced contaminant concentrations, and maintenance of functionality. Size-exclusion chromatography is particularly advantageous as it uses gravity, resulting in the preservation of EV structure, integrity, and biological activity. However, the extended isolation time represents a significant drawback for clinical studies, especially if high throughput sample processing is required (37).

Immunoaffinity (IA) purification is the least prevalent method of EV isolation, typically involving magnetic microbeads coated with an antibody that recognizes surface markers on the EV surface. While in principle IA represents a specific means for the identification and isolation of specific EVs, the lack of established and well-characterized EV markers limits its validity and utility. This method is additionally limited by the physical surface area of EVs available for binding. While potentially resulting in lower yields with higher purity, it may, however, result in concentration underestimations and false negative results (32, 37). While all the aforementioned techniques can be utilized to isolate EVs from culture media as well as biofluids, considerable care and caution should be exercised during optimization to ensure efficient enrichment. The lack of standardized isolation and characterization techniques has hindered advancement of the field. This is particularly evident when technique-to-technique comparisons are conducted, during which significantly different particle concentrations, characteristics, and functions can arise from biologically similar samples. Indeed, recent studies have suggested caution in the interpretation of EV investigations due to the likelihood of confounding factors, including co-purification of protein and lipid complexes (41, 42).

## EVs in Diabetic and Atherosclerotic Pathologies

**Figure 2:**
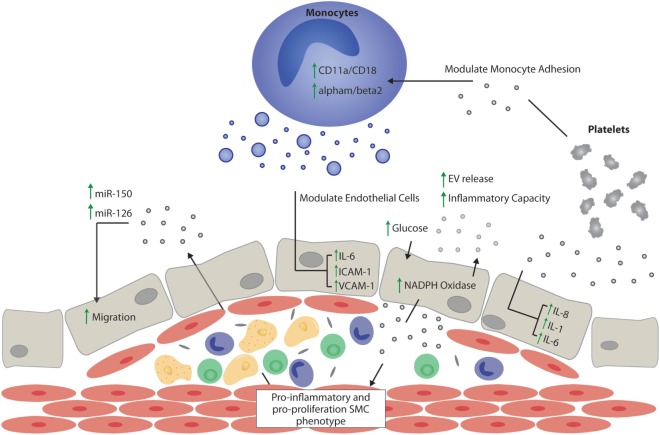
Extracellular vesicle (EV) effects on atherogenesis. Schematic representation of the potential proatherogenic and antiatherogenic effects of EVs, focusing mainly on the role of EVs in inflammation, thrombosis, and endothelial function. Vesicles of endothelial origin in the presence of hyperglycemia might stimulate pro-inflammatory and pro-proliferative smooth muscle cell phenotype switching. EVs stimulated by atherosclerotic plaque niche might stimulate or decrease vascular inflammation depending on their cargo proteins and noncoding RNAs. The presence of the miRNAs, miR-150, and miR-126, in endothelial vesicles is important in autoregulation of migration, while miR-150 is important in maintaining vascular smooth muscle cell differentiation. Vesicles of platelet origin promote endothelial and monocyte inflammation *via* interleukin (IL)-dependent mechanisms, and together with monocyte-derived vesicles, promote thrombosis by upregulating adhesion molecules. EVs released by monocytes contribute to endothelial inflammation by increasing leukocyte adhesion and activating the IL-6 pathway in endothelial cells. SMC, smooth muscle cell; interleukin-6, IL-6; interleukin-8, IL-8; interleukin-1, IL-1; intercellular adhesion molecule 1, ICAM-1; vascular cell adhesion molecule 1, VCAM-1.

Obstructive atherosclerotic diseases—disorders leading to the narrowing of the arterial lumen through the formation of atherosclerotic plaques—are thought to be central to the development of diabetic macrovascular complications (43). Metabolic dysfunction in individuals with T2DM has been shown to exacerbate and accelerate the pathological mechanisms underlying the development of atherosclerotic disease (44) (Figure 2). This is rooted in non-resolving proinflammatory pathogenic activation of the vascular endothelium; leading to platelet activation and adhesion, as well as the recruitment and trans-endothelial migration of circulating monocytes and neutrophils, which drive plaque expansion (45).

Endothelial cell (EC)-derived EVs have been described as important markers and mediators of vascular dysfunction. While patients with various types of vascular diseases have increases in levels of circulating EC-derived EVs, this is particularly evident in patients with both atherosclerosis and T2DM, who display markedly increased levels of circulating EC-derived EVs (46, 47). EVs appear to actively participate in the pathological progression of atherogenesis; from atherosclerotic lesion initiation to progression (48). For example, increased circulating levels of EC-derived EVs in T2DM appear to be associated with increased vascular dysfunction and are an independent risk factor for decreased arterial elasticity; a known change during atherogenesis (49). The decrease in arterial elasticity and successive development of high shear-stresses within the vasculature may further modulate EV function. In particular, platelet-derived EVs under high shear-stress conditions were shown to induce IL-8, IL-1β, and IL-6 production in ECs, which could indicate participation in vascular damage and atherosclerosis (50). Traditionally, the inflammatory response is mediated by the activation of the vascular endothelium and subsequent attraction of inflammatory cells, stimulation of the coagulation and complement systems, and increases in vascular permeability (51). Monocyte recruitment from the bloodstream represents one of the earliest processes of atherosclerotic plaque formation. While EVs isolated from healthy mouse plasma or endothelium can suppress monocyte activation, several experiments have shown that EVs isolated from activated ECs, platelets, or from atherosclerotic plaque, can promote the adhesion of monocytes to the endothelium by increasing the expression of adhesion molecules on both ECs and monocytes (52–54). Of note, Rautou et al. demonstrated that EVs isolated from symptomatic atherosclerotic plaques were more potent at promoting endothelial intercellular adhesion molecule 1-dependent monocyte adhesion and transendothelial migration than EVs from asymptomatic plaques (53). In addition, long-term feeding of high-fat diet to rats resulted in increased numbers of circulating EVs that were associated with an increased potential to induce pro-inflammatory reactive oxygen species and vascular cell adhesion molecule 1 expression in rat ECs *in vitro* (55). Similar observations have been made in the setting of hyperglycemia, where high-glucose conditions increased NADPH oxidase activity in endothelium-derived EVs, subsequently amplifying endothelial activation (56).

It is well established that phenotypic switching of smooth muscle cells has an important role in the progression of vascular diseases such as atherosclerosis (57). In the early stages of atherogenesis, smooth muscle cells acquire a synthetic phenotype and migrate from the media to the intima, subsequently proliferating and contributing to plaque development. Interestingly, platelet-derived EVs have been shown to actively induce vascular smooth muscle mitogenesis (58), while transfer of EVs from ECs to smooth muscle cells has been shown to either inhibit (59) or promote smooth muscle cell proliferation (60). In later stages of atherogenesis, microcalcification of vulnerable plaques can contribute to plaque destabilization and rupture (61). It is now evident that EVs play an active role in both the initiation and progression of calcification (62, 63). For example, New et al. provide clear support for the role of macrophage-derived EVs in the nucleation of microcalcifications (62).

Taken together, these results strongly suggest that EVs produced during the pathogenesis of diabetes and atherosclerosis not only promote the development of pro-inflammatory vascular conditions but also encourage the development of early atherosclerotic lesion development by promoting monocyte adhesion and infiltration to the sub-endothelial space, as well as through their ability to stimulate smooth muscle cell migration and proliferation and their role in instigating calcification. These findings provide unique insights into the pathogenesis and perhaps accelerated presentation of diabetes-associated atherogenesis.

Although there appears to be a strong relationship between EVs and atherogenesis, the exact functional interplay has yet to be fully explored. EV-associated miRNAs have received particular attention as they can be efficiently isolated from liquid biopsies and have substantial functional implications (64). Collectively, miRNAs have been shown to modulate vascular inflammatory, calcification, and thrombus formation pathways related to diabetes and atherosclerosis (65, 66). Jansen et al. described the differential regulation and selective packaging of miRNAs during T2DM pathology when compared to non-diabetic controls (67). Additionally, large-scale miRNA profiling of plasma EVs from patients with T2DM has revealed significant dysregulation of miRNAs, independent of body mass index, age, or sex (67). In-depth mechanistic studies have validated some functional roles of EV-associated miRNA dysregulation. Wu et al., in particular, found that the miRNA-126/VEGFR2 pathway was downregulated in untreated T2DM, potentially governing vascular integrity (68). Additionally, EC-EV transfer of miRNA-126 has been shown to be abrogated in high glucose settings, highlighting the importance of EV cargo maintenance in physiology (69). Karolina et al. has highlighted the promise of utilizing specific dysregulations in EV-associated miRNA cargo clinically by assessing the circulating EV-associated miRNA profiles of 219 participants with either metabolic syndrome, T2D, hypercholesterolemia, or hypertension, showing that each disorder had its own specific EV miRNA profile (70). While results of many studies, including the aforementioned one, have highlighted the unique dysregulation of EV-associated miRNA during the development of vascular disorders such as atherosclerosis, their utility as clinical biomarkers remain unfulfilled. This is in part a result of the complex and often multifactorial function of EVs, limiting our ability to efficiently delineate miRNAs directly associated with early disease processes.

## EVs in Diabetic Cardiac Pathology

Prolonged asymptomatic, yet progressive, phases of DCM make diagnosis of this condition particularly challenging (71). DCM is a complex condition and is defined as the presence of left ventricular (LV) dysfunction in individuals with T2DM in the absence of arterial hypertension, coronary artery disease, or evidence of other structural cardiac disease (71). While T2DM is a well-known risk factor for atherosclerotic disease, its role in development of DCM is less established. Epidemiological evidence suggests a high prevalence (30–40%) of cardiomyopathy in individuals with T2DM (72–75). Perhaps unsurprisingly, several signaling pathways (including inflammation, oxidative stress, and endothelial dysfunction) that are dysregulated under diabetic conditions and contribute to atherosclerotic disease also appear to enhance myocardial dysfunction (i.e., DCM) and accelerate heart failure (76–78). Clinically, DCM begins by presenting itself as early stage DCM, characterized by an abnormal myocardial energy metabolism, systolic, or diastolic dysfunction (i.e., impairment of the contraction of relaxation of the heart, respectively) and reduced LV strain (defined as regional deformation, or lengthening, shortening and thickening of the LV) (79). Over time, the progression of DCM can lead to overt heart failure, associated with cardiomyocyte hypertrophy, myocardial fibrosis, and ultimately cardiomyocyte death (80). This vulnerability to DCM may in part be due to the convergence of multiple risk factors, such as chronic hyperglycemia, resulting in detrimental effects on various cell types within the heart (81).

Recent attention has been focused on understanding the mechanisms of communication between the diverse cell-types in the heart, particularly, as it relates to disease pathogenesis (Table 2). These cells include cardiomyocytes (CMs), accounting for 25–35% of all cells in the heart (82), ECs (comprising 60% of the non-myocyte cells cardiac tissue cells), smooth muscle cells, hematopoietic-derived cells, and fibroblast cells (83). Each of these cell types play an important role in healthy and diseased cardiac function as they can contribute to the processes of ventricular hypertrophy, steatosis, fibrosis, and impaired angiogenesis, all of which can lead to diabetic cardiac complications, including cardiomyopathy (81). Extensive cross-talk occurs among these cells, and emerging evidence has implicated EVs in this communication (84). That being said, the link between EVs produced under T2DM conditions and increases in cardiac oxidative stress, cardiac inflammation, myocardial fibrosis, and other aspects of the pathogenesis of cardiomyopathy, has not been extensively studied to date, and remains an area ripe for future studies.

**Table 2:** Extracellular vesicle-derived miRNA regulation of the diabetic heart promotes the development of diabetic cardiomyopathies.

miRNA	Source/recipient	Target/process
miRNA-1/miRNA-133A	Cardiomyocytes (CMs)	Independent predictors of myocardial steatosis (85)
miRNA-320	CMs/endothelial cells (ECs)	Impairs angiogenesis by targeting IGF1, Hsp20, and Ets2 (86)
miRNA-503	ECs/pericytes	Impairs migration and proliferation following its transfer to vascular pericytes (87)
miRNA-126	Endothelial progenitor cells	Alters EC repair processes; reduces VEGFR-2 expression (68)
miRNA-21*	Cardiac fibroblasts/CMs	Promotes cardiac hypertrophy by targeting Sorbin and SH3-domain-containing protein 2 and PDLIM5 (88)

The main function of CMs is to generate contractile force in the heart, and although not considered to be a secretory cell, they can secrete cytokines, chemokines, and various factors such as ANP, and BNP as well as EVs (84). CM-derived EVs have been implicated in diabetic cardiomyocyte steatosis (85). Accumulation of lipids in the myocardium has been associated with non-ischemic cardiomyopathy (including DCM) and LV hypertrophy. Elevated levels of miR-1 and miR-133a were observed in EVs derived from lipid-loaded HL-1 CMs; levels were also increased in the serum of mice fed a high fat diet, and in the circulation of diabetic patients with myocardial steatosis (85). Unfortunately, no mechanism for miR-1/miR-133a function in steatosis was described, but being identified as independent predictors makes them important biomarkers. Recently, CM-derived EVs were shown to communicate with the endothelium, and it was demonstrated that this cross-talk is altered in the setting of diabetes; contributing to dysfunctional angiogenesis in DCM (86). While cardiomyocyte-derived EVs isolated from wild-type mice promoted angiogenesis, EVs isolated from diabetic rats exerted antiangiogenic effects; this was attributed to higher levels of antiangiogenic miR-320, and lower levels of angiogenic miR-126 (86).

Endothelial cells play a critical role in facilitating myocardial contraction and CM survival (89). Microvascular rarefaction is a major manifestation of diabetes-mediated ischemic cardiovascular disease, resulting from endothelial cell death and insufficient myocardial angiogenesis (90). Early in diabetes, high blood glucose leads to endothelial dysfunction, which can promote microvascular rarefaction over time (91). Several miRNA-based mechanisms have been proposed to explain vascular dysfunction in diabetes. For example, when exposed to elevated glucose concentrations, the levels of miR-503 in the endothelium increase, inhibiting EC proliferation and angiogenesis by targeting CCNE1 and Cdc25A (92). Moreover, transfer of miRNA-503 from EC-derived EVs impaired pericyte migration and proliferation, thereby decreasing angiogenesis and modulating vessel permeability by interfering with the production of VEGFA and EFNB2 (87). In healthy conditions, ECs release EVs that contain miR-10a, which can be transferred to monocytes, where it represses several components of the NF-κB signaling pathway to dampen their inflammatory activation (54). These EVs additionally contain high levels of miR-126, which can promote vascular endothelial repair through the targeting of SPRED-1 (69), a negative regulator of the VEGF signaling pathway (93, 94). However, in pathological hyperglycemic conditions, miR-126 expression is reduced in EC-EVs, impairing EC repair due to a lack of SPRED-1 targeting (69). Another group revealed reduced miR-126 expression in circulating EVs and endothelial progenitor cell-derived-EVs from patients with uncontrolled diabetes (68). Furthermore, exposing endothelial progenitor cells to these EVs downregulated VEGFR2 decreased migration ability, and increased apoptosis and ROS production (68).

Cardiac fibroblasts are involved in the fibrotic response that accompanies DCM. The differentiation of fibroblasts to myofibroblasts, together with their proliferation and production of extracellular matrix, contributes to the increased stiffness of the myocardium that promotes diastolic dysfunction (95). In neonatal rat cell culture, paracrine factors from cardiac fibroblasts elicit detrimental changes in CM electrophysiology that resemble those seen in cardiac pathologies (96); however, the role of EVs was not assessed. Hyperglycemia contributes to diabetic cardiac fibrosis as it can promote proliferation, myofibroblast differentiation, and collagen synthesis by cardiac fibroblasts (97–99). A potential culprit for the observed effects is miR-21, which targets DUSP5, a negative regulator of p38 and JNK signaling (100). Cardiac fibroblasts also secrete EVs that target CMs and appear to be enriched in miRNA passenger strands, which are typically eliminated during miRNA biogenesis. Transfer of miR-21* from cardiac fibroblasts to CMs induced cardiac hypertrophy by downregulating Sorbin and SH3-domain-containing protein 2 and PDZ and LIM domain 5 (PDLIM5) (88). Inhibition of miRNA-21* in mice with angiotensin II-induced heart hypertrophy suppressed the observed cardiac pathology (88).

Secretion of cardiac EVs appears to be an intricately regulated process that can mediate both local and systemic effects. *In vitro* cellular stretch and *in vivo* pressure overload in a mouse model induced the release of EVs from CMs that were enriched with angiotensin type I receptor (AT1R) (101). This was associated with the transfer of active AT1R to various tissues including the mesenteric artery and skeletal muscle, which upon injection into AT1 knockout mice, affected peripheral vascular resistance and blood pressure (101). The full extent of EV-mediated cell–cell communication among the cells locally in the heart or distally in systemic circulation has not yet been explored, and whether circulating EVs can be taken up by CMs, pericytes, or fibroblasts in the heart is not known. Additionally, the impact of diabetes on this form of communication is just coming into view. From initial studies, it appears that EVs are major protagonists in eliciting cardiovascular dysfunction in diabetics. Further elucidation of these pathways and mechanisms may reveal novel biomarkers and potential therapeutic strategies.

## Diabetic Cerebrovascular Cross Talk

Cardiovascular dysfunction, especially overt heart failure, has been proposed as a major cause of cognitive dysfunction in the elderly; commonly referred to as “vascular dementia” (102). An increasing body of evidence suggests that even the relatively mild effects on cardiac output that are observed in DCM are independently associated with impairment in various cognitive domains (103). Diabetes is associated with a breakdown in the blood brain barrier, a unique structure that protects the brain from detrimental systemic circulating factors (104). It is currently unclear whether cardiac output directly impacts cognitive function or whether both of these phenomena are driven by an independent factor. Based on the current body of evidence highlighting deleterious effects of inflammatory EVs on vascular ECs, it would seem conceivable to hypothesize that these effects on the brain and heart vasculature may be mediated by circulating EVs. Indeed, while still emerging, there is a body of evidence suggesting that EVs in diabetic microvascular settings may increase blood–brain barrier permeability (105, 106). A recent study found that the anti-inflammatory miRNA, miR-146a is decreased in the brains of diabetic mice, and that this is associated with accumulation of cellular prion protein (107). Interestingly, delivery of EC-derived exosomes loaded with miR-146a could decrease levels of cellular prion protein and could restore short-term memory (107). Additional research in this emerging area is clearly warranted and may shed light on the pathobiology of vascular dementia and its link to cardiac disease.

## EVs as Biomarkers of Diabetic Cardiovascular Pathologies

The paucity of effective diagnostic modalities and pharmacological interventions for DCM has fueled the search for novel circulating biomarkers that may be more reflective of disease status (108). Given their abundance in multiple bodily fluids and the modulation of the abundance, source, and contents (e.g., miRNAs) of EVs in response to pathological stimuli, EVs are attractive candidates as biomarkers (109). While numerous studies are underway to examine the utility of EVs as biomarkers (110–112), there has been a particularly intensive focus in oncological and neurological diseases. Understanding changes in EV contents will generate insight into potential disease mechanisms mediated by cell–cell communication that can be targeted therapeutically.

The complexity and chronic nature of cardiovascular pathologies appear to have impeded the field’s ability to correlate disease states with unique EV changes, slowing their adoption into biomarker studies. Nonetheless, there is considerable excitement in utilizing EVs as a novel diagnostic tool due to their inherent ability to transport miRNAs. miR-146a, in particular, may play an important role in the pathogenesis of both atherosclerosis (54, 113, 114) as well as the development of cardiomyopathies (115, 116) through the regulation of inflammatory pathways. Interestingly, it appears that transfer of miR-146a between cells may play an important role. For example, transfer between ECs and CMs plays a role in peripartum cardiomyopathy, and blocking this communication reverses pathology (115). In addition, the demonstration that miRNA-containing EVs are released into circulation from cardiac cells highlights the need for additional investigation (117). Identification of the cellular source of these EV-derived miRNA, understanding the mechanisms of packaging and secretion, and characterizing their functional roles remain a matter of active investigation. Nonetheless, a detailed characterization of EVs released into the circulation by CMs has lagged and appears prime for fruitful investigation.

## Therapeutic Potential of EVs

The involvement of EVs in the pathology of diabetic cardiovascular pathologies serves as a strong impetus to develop EV-based therapeutics. The combination of innate biocompatibility, low toxicity and immunogenicity, stability, and selective uptake make them an ideal delivery vehicle for therapeutics (118). Current therapeutic approaches aim to use EVs to deliver small RNAs in an attempt to reverse pathological miRNA-based communication with anti-miRNA oligonucleotides or to stimulate protective communication with synthetic miRNA mimics (119, 120). More specific delivery of anti-miRNAs or miRNA mimics to target cells is being achieved by engineering vesicles with cell-selective surface proteins (121), which should reduce off-target effects.

Many hurdles remain to be overcome before EV-based therapeutics might be used in the clinic to treat cardiovascular diseases. Nevertheless, the proven utility of using small RNAs in a cardioprotective manner in mouse and large animal models to prevent pathological changes such as fibrosis, cardiac hypertrophy and inflammation (122–124) highlights their potential as efficacious therapeutic targets. The ability to load EVs with particular cargo such as miRNAs, suggests the possibility of using EVs to deliver miRNA-based cardiovascular therapeutics. The field of miRNA-based therapeutics is advancing rapidly and over the last 10 years, research focused on circulating EVs, and the miRNA they contain, has revealed diverse and important roles (24). That being said, much still remains to be revealed regarding the role of EVs in cell–cell communication in health and diabetic cardiovascular disorders. Specifically, it may be advantageous to understand the effects of the chronic inflammatory environment in diabetes on the packaging and release of endothelial EVs and their subsequent interactions with CMs. Better understanding the role of endothelial-derived EVs may allow for in-depth probing of currently employed diabetes therapeutics such as sodium-glucose cotransporter-2 inhibitors, which are believed to have cardioprotective benefits (125). Advancing our understanding of the role of EVs in cardiovascular disease will help identify the cellular source and destination of EVs, subsequently allowing for the exploration of specific cellular interactions. Furthermore, improving our understanding of EV organ-tropism will aid in the targeting of specific tissues, improving the efficiency of miRNA-based therapies.

## Conclusion

Extracellular vesicles in liquid biopsies, such as blood, urine, or saliva, as well as localized tissue EV content remain a relatively untapped source of detailed information for both basic researchers and clinicians alike. The innate ability of EVs to shield biologically complex information from degradation and sensitivity to minute changes in physiology highlight their potential as sensitive and specific biomarkers. Early studies into their biology suggest that they may be critical mediators of cardiovascular diseases such as atherosclerosis and DCM. There are a number of unexplored avenues particularly regarding the interactions between elevated glucose levels, endothelial EVs, and dysfunction in cardiac tissues. Understanding the potential roles of EVs in diabetes associated cardiac dysfunction will be critical in understanding the mechanisms of currently employed therapeutics and for the development of more efficacious agents. The largest roadblock in illuminating the roles of EVs in the cardiovascular field remains a thorough understanding of the vesicle population, which in suit relies heavily upon our ability to apply accurate vesicle isolation techniques. The development of a harmonized nomenclature for EVs will be essential for both meaningful dialog between researchers and ensuring reproducibility of results across laboratories (109). To better understand the role of EVs in multifactorial conditions such as diabetic pathologies, a number of gaps in fundamental knowledge should be addressed. The most pressing is to better understand the mechanisms of EV biogenesis, delivery, and degradation upon which a more normalized nomenclature can be developed. Building upon this, the development of accurate *in vivo* vesicle tracking models will be essential in validating much of what is currently known for translation into the clinic. Finally, utilizing large clinical cohorts for the examination of vesicle concentrations, populations, and cargo should be performed to examine vesicle heterogeneity in multiple patient populations. Although the precise physiological and pathological functions of EVs remain at a nascent stage of understanding, their obvious potential as biomarkers and vehicles for therapeutic intervention could transform our approach to understanding and treating diabetic cardiovascular pathologies.

## Author Contributions

DG and SV researched the data for the article, substantially contributed to discussion of the article and wrote the article. DG, SV, and JF contributed to conceptualizing the article as well as reviewing and editing of the manuscript before submission.

## Conflict of Interest Statement

The authors declare that the research was conducted in the absence of any commercial or financial relationships that could be construed as a potential conflict of interest.

## Abbreviations

CM, cardiomyocytes; DCM, diabetic cardiomyopathy; DM, diabetes mellitus; ECs, endothelial cells; EVs, extracellular vesicles; mRNA, messenger ribonucleic acid; MVs, microvesicles; MVBs, multivesicular bodies; T2DM, type 2 diabetes mellitus.

## References

[1] Shaw JE Sicree RA Zimmet PZ. Global estimates of the prevalence of diabetes for 2010 and 2030. Diabetes Res Clin Pract (2010) 87(1):4–14. 10.1016/j.diabres.2009.10.007 19896746

[2] Guariguata L Whiting D Hambleton I Beagley J Linnenkamp U Shaw J. Global estimates of diabetes prevalence for 2013 and projections for 2035. Diabetes Res Clin Pract (2014) 103(2):137–49. 10.1016/j.diabres.2013.11.002 24630390

[3] Tancredi M Rosengren A Svensson A-M Kosiborod M Pivodic A Gudbjörnsdottir S Excess mortality among persons with type 2 diabetes. N Engl J Med (2015) 373(18):1720–32. 10.1056/NEJMoa1504347 26510021

[4] Mazzone T Chait A Plutzky J. Cardiovascular disease risk in type 2 diabetes mellitus: insights from mechanistic studies. Lancet (2008) 371(9626):1800–9. 10.1016/S0140-6736(08)60768-0 18502305 PMC2774464

[5] Wang CCL Hess CN Hiatt WR Goldfine AB Clinical update: cardiovascular disease in diabetes mellitus. Circulation (2016) 133(24):2459–502. 10.1161/CIRCULATIONAHA.116.022194 27297342 PMC4910510

[6] Shah AD Langenberg C Rapsomaniki E Denaxas S Pujades-Rodriguez M Gale CP Type 2 diabetes and incidence of cardiovascular diseases: a cohort study in 1·9 million people. Lancet Diabetes Endocrinol (2015) 3(2):105–13. 10.1016/S2213-8587(14)70219-0 25466521 PMC4303913

[7] Fowler MJ Microvascular and macrovascular complications of diabetes. Clin Diabetes (2008) 26(2):77–82. 10.2337/diaclin.26.2.77

[8] Yahagi K Kolodgie FD Lutter C Mori H Romero ME Finn AV Pathology of human coronary and carotid artery atherosclerosis and vascular calcification in diabetes mellitus. Arterioscler Thromb Vasc Biol (2017) 37(2):191–204. 10.1161/atvbaha.116.306256 27908890 PMC5269516

[9] Raposo G Stoorvogel W. Extracellular vesicles: exosomes, microvesicles, and friends. J Cell Biol (2013) 200(4):373–83. 10.1083/jcb.201211138 23420871 PMC3575529

[10] Pol E Böing A Gool E Nieuwland R. Recent developments in the nomenclature, presence, isolation, detection and clinical impact of extracellular vesicles. J Thromb Haemost (2016) 14(1):48–56. 10.1111/jth.13190 26564379

[11] Kowal J Tkach M Théry C. Biogenesis and secretion of exosomes. Curr Opin Cell Biol (2014) 29:116–25. 10.1016/j.ceb.2014.05.004 24959705

[12] Henne WM Stenmark H Emr SD. Molecular mechanisms of the membrane sculpting ESCRT pathway. Cold Spring Harb Perspect Biol (2013) 5(9):a016766. 10.1101/cshperspect.a016766 24003212 PMC3753708

[13] Klumperman J Raposo G. The complex ultrastructure of the endolysosomal system. Cold Spring Harb Perspect Biol (2014) 6(10):a016857. 10.1101/cshperspect.a016857 24851870 PMC4176003

[14] Trajkovic K Hsu C Chiantia S Rajendran L Wenzel D Wieland F Ceramide triggers budding of exosome vesicles into multivesicular endosomes. Science (2008) 319(5867):1244–7. 10.1126/science.1153124 18309083

[15] van Niel G Charrin S Simoes S Romao M Rochin L Saftig P The tetraspanin CD63 regulates ESCRT-independent and dependent endosomal sorting during melanogenesis. Dev Cell (2011) 21(4):708–21. 10.1016/j.devcel.2011.08.019 21962903 PMC3199340

[16] Hsu C Morohashi Y Yoshimura S-I Manrique-Hoyos N Jung S Lauterbach MA Regulation of exosome secretion by Rab35 and its GTPase-activating proteins TBC1D10A–C. J Cell Biol (2010) 189(2):223–32. 10.1083/jcb.200911018 20404108 PMC2856897

[17] Cocucci E Racchetti G Meldolesi J Shedding microvesicles: artefacts no more. Trends Cell Biol (2009) 19(2):43–51. 10.1016/j.tcb.2008.11.003 19144520

[18] Del Conde I Shrimpton CN Thiagarajan P Lopez JA. Tissue-factor-bearing microvesicles arise from lipid rafts and fuse with activated platelets to initiate coagulation. Blood (2005) 106(5):1604–11. 10.1182/blood-2004-03-1095 15741221

[19] Hugel B Martinez MC Kunzelmann C Freyssinet JM. Membrane microparticles: two sides of the coin. Physiology (Bethesda) (2005) 20:22–7. 10.1152/physiol.00029.2004 15653836

[20] Kerr JF Wyllie AH Currie AR. Apoptosis: a basic biological phenomenon with wide-ranging implications in tissue kinetics. Br J Cancer (1972) 26(4):239–57. 10.1038/bjc.1972.33 4561027 PMC2008650

[21] Elmore S Apoptosis: a review of programmed cell death. Toxicol Pathol (2007) 35(4):495–516. 10.1080/01926230701320337 17562483 PMC2117903

[22] Mulcahy LA Pink RC Carter DRF. Routes and mechanisms of extracellular vesicle uptake. J Extracell Vesicles (2014):3. 10.3402/jev.v3403.24641 25143819 PMC4122821

[23] Maas SLN Breakefield XO Weaver AM. Extracellular vesicles: unique intercellular delivery vehicles. Trends Cell Biol (2017) 27(3):172–88. 10.1016/j.tcb.2016.11.003 27979573 PMC5318253

[24] El Andaloussi S Mager I Breakefield XO Wood MJA Extracellular vesicles: biology and emerging therapeutic opportunities. Nat Rev Drug Discov (2013) 12(5):347–57. 10.1038/nrd3978 23584393

[25] Ramakrishnan DP Hajj-Ali RA Chen Y Silverstein RL Extracellular vesicles activate a CD36-dependent signaling pathway to inhibit microvascular endothelial cell migration and tube formation significance. Arterioscler Thromb Vasc Biol (2016) 36(3):534–44. 10.1161/ATVBAHA.115.307085 26821945 PMC4767682

[26] Cambier L de Couto G Ibrahim A Echavez AK Valle J Liu W Y RNA fragment in extracellular vesicles confers cardioprotection via modulation of IL-10 expression and secretion. EMBO Mol Med (2017) 9(3):337–52. 10.15252/emmm.201606924 28167565 PMC5331234

[27] Meister G Tuschi T. Mechanisms of gene silencing by double-stranded RNA. Nature (2004) 431(7006):343. 10.1038/nature02873 15372041

[28] Lázaro-Ibáñez E Sanz-Garcia A Visakorpi T Escobedo-Lucea C Siljander P Ayuso-Sacido Á Different gDNA content in the subpopulations of prostate cancer extracellular vesicles: apoptotic bodies, microvesicles, and exosomes. Prostate (2014) 74(14):1379–90. 10.1002/pros.22853 25111183 PMC4312964

[29] Haraszti RA Didiot MC Sapp E Leszyk J Shaffer SA Rockwell HE High-resolution proteomic and lipidomic analysis of exosomes and microvesicles from different cell sources. J Extracell Vesicles (2016) 5:32570. 10.3402/jev.v5.32570 27863537 PMC5116062

[30] Zernecke A Bidzhekov K Noels H Shagdarsuren E Gan L Denecke B Delivery of microRNA-126 by apoptotic bodies induces CXCL12-dependent vascular protection. Sci Signal (2009) 2(100):ra81. 10.1126/scisignal.2000610 19996457

[31] Zarovni N Corrado A Guazzi P Zocco D Lari E Radano G Integrated isolation and quantitative analysis of exosome shuttled proteins and nucleic acids using immunocapture approaches. Methods (2015) 87:46–58. 10.1016/j.ymeth.2015.05.028 26044649

[32] Li P Kaslan M Lee SH Yao J Gao Z. Progress in exosome isolation techniques. Theranostics (2017) 7(3):789–804. 10.7150/thno.18133 28255367 PMC5327650

[33] Thery C Amigorena S Raposo G Clayton A Isolation and characterization of exosomes from cell culture supernatants and biological fluids. Curr Protoc Cell Biol (2006) Chapter 3:Unit 3.22. 10.1002/0471143030.cb0322s30 18228490

[34] Baran J Baj-Krzyworzeka M Weglarczyk K Szatanek R Zembala M Barbasz J Circulating tumour-derived microvesicles in plasma of gastric cancer patients. Cancer Immunol Immunother (2010) 59(6):841–50. 10.1007/s00262-009-0808-2 20043223 PMC11030063

[35] Witwer KW Buzas EI Bemis LT Bora A Lasser C Lotvall J Standardization of sample collection, isolation and analysis methods in extracellular vesicle research. J Extracell Vesicles (2013) 2 10.3402/jev.v2i0.20360 PMC3760646 24009894

[36] Jeppesen DK Hvam ML Primdahl-Bengtson B Boysen AT Whitehead B Dyrskjot L Comparative analysis of discrete exosome fractions obtained by differential centrifugation. J Extracell Vesicles (2014) 3:25011. 10.3402/jev.v3.25011 25396408 PMC4224706

[37] Zeringer E Barta T Li M Vlassov AV Strategies for isolation of exosomes. Cold Spring Harb Protoc (2015) 2015(4):319–23. 10.1101/pdb.top074476 25834266

[38] Taylor DD Zacharias W Gercel-Taylor C. Exosome isolation for proteomic analyses and RNA profiling. Methods Mol Biol (2011) 728:235–46. 10.1007/978-1-61779-068-3_15 21468952

[39] Heinemann ML Ilmer M Silva LP Hawke DH Recio A Vorontsova MA Benchtop isolation and characterization of functional exosomes by sequential filtration. J Chromatogr A (2014) 1371:125–35. 10.1016/j.chroma.2014.10.026 25458527

[40] Gamez-Valero A Monguio-Tortajada M Carreras-Planella L Franquesa M Beyer K Borras FE. Size-exclusion chromatography-based isolation minimally alters extracellular vesicles’ characteristics compared to precipitating agents. Sci Rep (2016) 6:33641. 10.1038/srep33641 27640641 PMC5027519

[41] Erdbrügger U Lannigan J. Analytical challenges of extracellular vesicle detection: a comparison of different techniques. Cytometry A (2016) 89(2):123–34. 10.1002/cyto.a.22795 26651033

[42] Tosar JP Cayota A Eitan E Halushka MK Witwer KW. Ribonucleic artefacts: are some extracellular RNA discoveries driven by cell culture medium components? J Extracell Vesicles (2017) 6(1):1272832. 10.1080/20013078.2016.1272832 28326168 PMC5328325

[43] Rahman S Rahman T Ismail AA-S Rashid ARA. Diabetes-associated macrovasculopathy: pathophysiology and pathogenesis. Diabetes Obes Metab (2007) 9(6):767–80. 10.1111/j.1463-1326.2006.00655.x 17924861

[44] Bornfeldt KE Tabas I Insulin resistance, hyperglycemia, and atherosclerosis. Cell Metab (2011) 14(5):575–85. 10.1016/j.cmet.2011.07.015 22055501 PMC3217209

[45] Galkina E Ley K Immune and inflammatory mechanisms of atherosclerosis. Annu Rev Immunol (2009) 27:165–97. 10.1146/annurev.immunol.021908.132620 19302038 PMC2734407

[46] Koga H Sugiyama S Kugiyama K Watanabe K Fukushima H Tanaka T Elevated levels of VE-cadherin-positive endothelial microparticles in patients with type 2 diabetes mellitus and coronary artery disease. J Am Coll Cardiol (2005) 45(10):1622–30. 10.1016/j.jacc.2005.02.047 15893178

[47] Tramontano AF Lyubarova R Tsiakos J Palaia T Deleon JR Ragolia L Circulating endothelial microparticles in diabetes mellitus. Mediators Inflamm (2010) 2010:250476 10.1155/2010/250476 20634911 PMC2904448

[48] Aurelian SM Cheta DM Onicescu D Microvesicles – potential biomarkers for the interrelations atherosclerosis/type 2 diabetes mellitus. Rom J Morphol Embryol (2014) 55(3 Suppl):1035–9. 25607382

[49] Feng B Chen Y Luo Y Chen M Li X Ni Y. Circulating level of microparticles and their correlation with arterial elasticity and endothelium-dependent dilation in patients with type 2 diabetes mellitus. Atherosclerosis (2010) 208(1):264–9. 10.1016/j.atherosclerosis.2009.06.037 19674745

[50] Nomura S Tandon NN Nakamura T Cone J Fukuhara S Kambayashi J. High-shear-stress-induced activation of platelets and microparticles enhances expression of cell adhesion molecules in THP-1 and endothelial cells. Atherosclerosis (2001) 158(2):277–87. 10.1016/S0021-9150(01)00433-6 11583705

[51] Libby P History of discovery: inflammation in atherosclerosis. Arterioscler Thromb Vasc Biol (2012) 32(9):2045–51. 10.1161/ATVBAHA.108.179705 22895665 PMC3422754

[52] Barry OP Praticò D Savani RC FitzGerald GA. Modulation of monocyte-endothelial cell interactions by platelet microparticles. J Clin Invest (1998) 102(1):136. 10.1172/JCI2592 9649567 PMC509075

[53] Rautou PE Leroyer AS Ramkhelawon B Devue C Duflaut D Vion AC Microparticles from human atherosclerotic plaques promote endothelial ICAM-1-dependent monocyte adhesion and transendothelial migration. Circ Res (2011) 108(3):335–43. 10.1161/circresaha.110.237420 21164106

[54] Njock M-S Cheng HS Dang LT Nazari-Jahantigh M Lau AC Boudreau E Endothelial cells suppress monocyte activation through secretion of extracellular vesicles containing anti-inflammatory microRNAs. Blood (2015) 125(20):3202–12. 10.1182/blood-2014-11-611046 25838349 PMC4440888

[55] Heinrich LF Andersen DK Cleasby ME Lawson C. Long-term high fat feeding of rats results in increased numbers of circulating microvesicles with pro-inflammatory effects on endothelial cells. Br J Nutr (2015) 113(11):1704–11. 10.1017/s0007114515001117 25880162

[56] Jansen F Yang X Franklin BS Hoelscher M Schmitz T Bedorf J High glucose condition increases NADPH oxidase activity in endothelial microparticles that promote vascular inflammation. Cardiovasc Res (2013) 98(1):94–106. 10.1093/cvr/cvt013 23341580

[57] Gomez D Owens GK. Smooth muscle cell phenotypic switching in atherosclerosis. Cardiovasc Res (2012) 95(2):156–64. 10.1093/cvr/cvs115 22406749 PMC3388816

[58] Weber A-A Köppen HO Schrör K. Platelet-derived microparticles stimulate coronary artery smooth muscle cell mitogenesis by a PDGF-independent mechanism. Thromb Res (2000) 98(5):461–6. 10.1016/S0049-3848(00)00192-4 10828486

[59] Jansen F Stumpf T Proebsting S Franklin BS Wenzel D Pfeifer P Intercellular transfer of miR-126-3p by endothelial microparticles reduces vascular smooth muscle cell proliferation and limits neointima formation by inhibiting LRP6. J Mol Cell Cardiol (2017) 104:43–52. 10.1016/j.yjmcc.2016.12.005 28143713

[60] Zhou J Li YS Nguyen P Wang KC Weiss A Kuo YC Regulation of vascular smooth muscle cell turnover by endothelial cell-secreted microRNA-126: role of shear stress. Circ Res (2013) 113(1):40–51. 10.1161/circresaha.113.280883 23603512 PMC3772783

[61] Bobryshev Y Killingsworth M Lord R Grabs A. Matrix vesicles in the fibrous cap of atherosclerotic plaque: possible contribution to plaque rupture. J Cell Mol Med (2008) 12(5b):2073–82. 10.1111/j.1582-4934.2008.00230.x 18194456 PMC4506172

[62] New SE Goettsch C Aikawa M Marchini JF Shibasaki M Yabusaki K Macrophage-derived matrix vesicles: an alternative novel mechanism for microcalcification in atherosclerotic plaques. Circ Res (2013) 113(1):72–7. 10.1161/CIRCRESAHA.113.301036 23616621 PMC3703850

[63] Goettsch C Hutcheson JD Aikawa M Iwata H Pham T Nykjaer A Sortilin mediates vascular calcification via its recruitment into extracellular vesicles. J Clin Invest (2016) 126(4):1323–36. 10.1172/JCI80851 26950419 PMC4811143

[64] Hunter MP Ismail N Zhang X Aguda BD Lee EJ Yu L Detection of microRNA expression in human peripheral blood microvesicles. PLoS One (2008) 3(11):e3694. 10.1371/journal.pone.0003694 19002258 PMC2577891

[65] Cheng HS Sivachandran N Lau A Boudreau E Zhao JL Baltimore D MicroRNA-146 represses endothelial activation by inhibiting pro-inflammatory pathways. EMBO Mol Med (2013) 5(7):949–66. 10.1002/emmm.201202318 23733368 PMC3721471

[66] Das S Halushka MK Extracellular vesicle microRNA transfer in cardiovascular disease. Cardiovasc Pathol (2015) 24(4):199–206. 10.1016/j.carpath.2015.04.007 25958013

[67] Jansen F Wang H Przybilla D Franklin BS Dolf A Pfeifer P Vascular endothelial microparticles-incorporated microRNAs are altered in patients with diabetes mellitus. Cardiovasc Diabetol (2016) 15:49. 10.1186/s12933-016-0367-8 27005938 PMC4804519

[68] Wu K Yang Y Zhong Y Ammar HM Zhang P Guo R The effects of microvesicles on endothelial progenitor cells are compromised in type 2 diabetic patients via downregulation of the miR-126/VEGFR2 pathway. Am J Physiol (2016) 310(10):E828–37. 10.1152/ajpendo.00056.2016 26956185 PMC4895450

[69] Jansen F Yang X Hoelscher M Cattelan A Schmitz T Proebsting S Endothelial microparticle-mediated transfer of microRNA-126 promotes vascular endothelial cell repair via SPRED1 and is abrogated in glucose-damaged endothelial microparticles. Circulation (2013) 128(18):2026–38. 10.1161/circulationaha.113.001720 24014835

[70] Karolina DS Tavintharan S Armugam A Sepramaniam S Pek SLT Wong MT Circulating miRNA profiles in patients with metabolic syndrome. J Clin Endocrinol Metabol (2012) 97(12):E2271–6. 10.1210/jc.2012-1996 23032062

[71] Elliott P Andersson B Arbustini E Bilinska Z Cecchi F Charron P Classification of the cardiomyopathies: a position statement from the European Society Of Cardiology Working Group on Myocardial and Pericardial Diseases. Eur Heart J (2008) 29(2):270–6. 10.1093/eurheartj/ehm342 17916581

[72] Cohn JN Johnson G Ziesche S Cobb F Francis G Tristani F A comparison of enalapril with hydralazine-isosorbide dinitrate in the treatment of chronic congestive heart failure. N Engl J Med (1991) 325(5):303–10. 10.1056/nejm199108013250502 2057035

[73] Shindler DM Kostis JB Yusuf S Quinones MA Pitt B Stewart D Diabetes mellitus, a predictor of morbidity and mortality in the Studies of Left Ventricular Dysfunction (SOLVD) Trials and Registry. Am J Cardiol (1996) 77(11):1017–20. 10.1016/S0002-9149(97)89163-1 8644628

[74] Ryden L Armstrong PW Cleland JG Horowitz JD Massie BM Packer M Efficacy and safety of high-dose lisinopril in chronic heart failure patients at high cardiovascular risk, including those with diabetes mellitus. Results from the ATLAS trial. Eur Heart J (2000) 21(23):1967–78. 10.1053/euhj.2000.2311 11071803

[75] Pappachan JM Varughese GI Sriraman R Arunagirinathan G. Diabetic cardiomyopathy: pathophysiology, diagnostic evaluation and management. World J Diabetes (2013) 4(5):177–89. 10.4239/wjd.v4.i5.177 24147202 PMC3797883

[76] Farhangkhoee H Khan ZA Kaur H Xin X Chen S Chakrabarti S. Vascular endothelial dysfunction in diabetic cardiomyopathy: pathogenesis and potential treatment targets. Pharmacol Ther (2006) 111(2):384–99. 10.1016/j.pharmthera.2005.10.008 16343639

[77] Palomer X Salvado L Barroso E Vazquez-Carrera M. An overview of the crosstalk between inflammatory processes and metabolic dysregulation during diabetic cardiomyopathy. Int J Cardiol (2013) 168(4):3160–72. 10.1016/j.ijcard.2013.07.150 23932046

[78] Liu Q Wang S Cai L Diabetic cardiomyopathy and its mechanisms: role of oxidative stress and damage. J Diabetes Investig (2014) 5(6):623–34. 10.1111/jdi.12250 PMC4234223 25422760

[79] Gorcsan J III Tanaka H Echocardiographic assessment of myocardial strain. J Am Coll Cardiol (2011) 58(14):1401–13. 10.1016/j.jacc.2011.06.038 21939821

[80] Westermeier F Riquelme JA Pavez M Garrido V Diaz A Verdejo HE New molecular insights of insulin in diabetic cardiomyopathy. Front Physiol (2016) 7:125. 10.3389/fphys.2016.00125 27148064 PMC4828458

[81] Voulgari C Papadogiannis D Tentolouris N. Diabetic cardiomyopathy: from the pathophysiology of the cardiac myocytes to current diagnosis and management strategies. Vasc Health Risk Manag (2010) 6:883–903. 10.2147/vhrm.s11681 21057575 PMC2964943

[82] Bergmann O Zdunek S Felker A Salehpour M Alkass K Bernard S Dynamics of cell generation and turnover in the human heart. Cell (2015) 161(7):1566–75. 10.1016/j.cell.2015.05.026 26073943

[83] Pinto AR Ilinykh A Ivey MJ Kuwabara JT D’Antoni M Debuque RJ Revisiting cardiac cellular composition. Circ Res (2015) 118(3):400–9. 10.1161/CIRCRESAHA.115.307778 26635390 PMC4744092

[84] Sluijter JP Verhage V Deddens JC van den Akker F Doevendans PA. Microvesicles and exosomes for intracardiac communication. Cardiovasc Res (2014) 102(2):302–11. 10.1093/cvr/cvu022 24488559

[85] de Gonzalo-Calvo D van der Meer RW Rijzewijk LJ Smit JW Revuelta-Lopez E Nasarre L Serum microRNA-1 and microRNA-133a levels reflect myocardial steatosis in uncomplicated type 2 diabetes. Sci Rep (2017) 7(1):47. 10.1038/s41598-017-00070-6 28246388 PMC5428350

[86] Wang X Huang W Liu G Cai W Millard RW Wang Y Cardiomyocytes mediate anti-angiogenesis in type 2 diabetic rats through the exosomal transfer of miR-320 into endothelial cells. J Mol Cell Cardiol (2014) 74:139–50. 10.1016/j.yjmcc.2014.05.001 24825548 PMC4120246

[87] Caporali A Meloni M Nailor A Mitic T Shantikumar S Riu F p75(NTR)-dependent activation of NF-kappaB regulates microRNA-503 transcription and pericyte-endothelial crosstalk in diabetes after limb ischaemia. Nat Commun (2015) 6:8024 10.1038/ncomms9024 26268439 PMC4538859

[88] Bang C Batkai S Dangwal S Gupta SK Foinquinos A Holzmann A Cardiac fibroblast-derived microRNA passenger strand-enriched exosomes mediate cardiomyocyte hypertrophy. J Clin Invest (2014) 124(5):2136–46. 10.1172/jci70577 24743145 PMC4001534

[89] Hsieh PC Davis ME Lisowski LK Lee RT. Endothelial-cardiomyocyte interactions in cardiac development and repair. Annu Rev Physiol (2006) 68:51–66. 10.1146/annurev.physiol.68.040104.124629 16460266 PMC2754585

[90] Varga ZV Giricz Z Liaudet L Hasko G Ferdinandy P Pacher P. Interplay of oxidative, nitrosative/nitrative stress, inflammation, cell death and autophagy in diabetic cardiomyopathy. Biochim Biophys Acta (2015) 1852(2):232–42. 10.1016/j.bbadis.2014.06.030 24997452 PMC4277896

[91] Nakagami H Kaneda Y Ogihara T Morishita R. Endothelial dysfunction in hyperglycemia as a trigger of atherosclerosis. Curr Diabetes Rev (2005) 1(1):59–63. 10.2174/1573399052952550 18220582

[92] Caporali A Meloni M Vollenkle C Bonci D Sala-Newby GB Addis R Deregulation of microRNA-503 contributes to diabetes mellitus-induced impairment of endothelial function and reparative angiogenesis after limb ischemia. Circulation (2011) 123(3):282–91. 10.1161/circulationaha.110.952325 21220732

[93] Fish JE Santoro MM Morton SU Yu S Yeh RF Wythe JD miR-126 regulates angiogenic signaling and vascular integrity. Dev Cell (2008) 15(2):272–84. 10.1016/j.devcel.2008.07.008 18694566 PMC2604134

[94] Wang S Aurora AB Johnson BA Qi X McAnally J Hill JA The endothelial-specific microRNA miR-126 governs vascular integrity and angiogenesis. Dev Cell (2008) 15(2):261–71. 10.1016/j.devcel.2008.07.002 18694565 PMC2685763

[95] Fan D Takawale A Lee J Kassiri Z Cardiac fibroblasts, fibrosis and extracellular matrix remodeling in heart disease. Fibrogenesis Tissue Repair (2012) 5(1):15 10.1186/1755-1536-5-15 22943504 PMC3464725

[96] Pedrotty DM Klinger RY Kirkton RD Bursac N. Cardiac fibroblast paracrine factors alter impulse conduction and ion channel expression of neonatal rat cardiomyocytes. Cardiovasc Res (2009) 83(4):688–97. 10.1093/cvr/cvp164 19477968 PMC2725777

[97] Shamhart PE Luther DJ Adapala RK Bryant JE Petersen KA Meszaros JG Hyperglycemia enhances function and differentiation of adult rat cardiac fibroblasts. Can J Physiol Pharmacol (2014) 92(7):598–604. 10.1139/cjpp-2013-0490 24959995 PMC4883005

[98] Chen X Liu G Zhang W Zhang J Yan Y Dong W Inhibition of MEF2A prevents hyperglycemia-induced extracellular matrix accumulation by blocking Akt and TGF-beta1/Smad activation in cardiac fibroblasts. Int J Biochem Cell Biol (2015) 69:52–61. 10.1016/j.biocel.2015.10.012 26482596

[99] Liu J Zhuo X Liu W Wan Z Liang X Gao S Resveratrol inhibits high glucose induced collagen upregulation in cardiac fibroblasts through regulating TGF-beta1-Smad3 signaling pathway. Chem Biol Interact (2015) 227:45–52. 10.1016/j.cbi.2014.12.031 25559857

[100] Liu S Li W Xu M Huang H Wang J Chen X Micro-RNA 21Targets dual specific phosphatase 8 to promote collagen synthesis in high glucose-treated primary cardiac fibroblasts. Can J Cardiol (2014) 30(12):1689–99. 10.1016/j.cjca.2014.07.747 25418215

[101] Pironti G Strachan RT Abraham D Mon-Wei Yu S Chen M Chen W Circulating exosomes induced by cardiac pressure overload contain functional angiotensin II type 1 receptors. Circulation (2015) 131(24):2120–30. 10.1161/circulationaha.115.015687 25995315 PMC4470842

[102] Roman GC Vascular dementia may be the most common form of dementia in the elderly. J Neurol Sci (2002) 203-204:7–10. 10.1016/S0022-510X(02)00252-6 12417349

[103] Vogels RL Scheltens P Schroeder-Tanka JM Weinstein HC Cognitive impairment in heart failure: a systematic review of the literature. Eur J Heart Fail (2007) 9(5):440–9. 10.1016/j.ejheart.2006.11.001 17174152

[104] Bogush M Heldt NA Persidsky Y Blood brain barrier injury in diabetes: unrecognized effects on brain and cognition. J Neuroimmune Pharmacol (2017). 10.1007/s11481-017-9752-7 PMC5693692 28555373

[105] Beltramo E Lopatina T Berrone E Mazzeo A Iavello A Camussi G Extracellular vesicles derived from mesenchymal stem cells induce features of diabetic retinopathy in vitro. Acta Diabetol (2014) 51(6):1055. 10.1007/s00592-014-0672-1 25374383

[106] Andras IE Toborek M Extracellular vesicles of the blood-brain barrier. Tissue Barriers (2016) 4(1):e1131804 10.1080/21688370.2015.1131804 27141419 PMC4836554

[107] Kalani A Chaturvedi P Maldonado C Bauer P Joshua IG Tyagi SC Dementia-like pathology in type-2 diabetes: a novel microRNA mechanism. Mol Cell Neurosci (2017) 80:58–65. 10.1016/j.mcn.2017.02.005 28219659 PMC5432966

[108] Sanders-van Wijk S van Empel V Davarzani N Maeder MT Handschin R Pfisterer ME Circulating biomarkers of distinct pathophysiological pathways in heart failure with preserved vs. reduced left ventricular ejection fraction. Eur J Heart Fail (2015) 17(10):1006–14. 10.1002/ejhf.414 26472682

[109] Boulanger CM Loyer X Rautou PE Amabile N. Extracellular vesicles in coronary artery disease. Nat Rev Cardiol (2017) 14(5):259–72. 10.1038/nrcardio.2017.7 28150804

[110] Santovito D De Nardis V Marcantonio P Mandolini C Paganelli C Vitale E Plasma exosome microRNA profiling unravels a new potential modulator of adiponectin pathway in diabetes: effect of glycemic control. J Clin Endocrinol Metab (2014) 99(9):E1681–5. 10.1210/jc.2013-3843 24937531

[111] Thompson AG Gray E Heman-Ackah SM Mager I Talbot K Andaloussi SE Extracellular vesicles in neurodegenerative disease – pathogenesis to biomarkers. Nat Rev Neurol (2016) 12(6):346–57. 10.1038/nrneurol.2016.68 27174238

[112] Wang J Yan F Zhao Q Zhan F Wang R Wang L Circulating exosomal miR-125a-3p as a novel biomarker for early-stage colon cancer. Sci Rep (2017) 7(1):4150. 10.1038/s41598-017-04386-1 28646161 PMC5482839

[113] Cheng HS Njock MS Khyzha N Dang LT Fish JE Noncoding RNAs regulate NF-kappaB signaling to modulate blood vessel inflammation. Front Genet (2014) 5:422 10.3389/fgene.2014.00422 25540650 PMC4261819

[114] Cheng HS Besla R Li A Chen Z Shikatani EA Nazari-Jahantigh M Paradoxical suppression of atherosclerosis in the absence of microRNA-146a. Circ Res (2017) 121(4):354–67. 10.1161/circresaha.116.310529 28637783 PMC5542783

[115] Halkein J Tabruyn SP Ricke-Hoch M Haghikia A Nguyen NQ Scherr M MicroRNA-146a is a therapeutic target and biomarker for peripartum cardiomyopathy. J Clin Invest (2013) 123(5):2143–54. 10.1172/jci64365 23619365 PMC3638905

[116] Feng B Chen S Gordon AD Chakrabarti S. miR-146a mediates inflammatory changes and fibrosis in the heart in diabetes. J Mol Cell Cardiol (2017) 105:70–6. 10.1016/j.yjmcc.2017.03.002 28279663

[117] De Rosa S Fichtlscherer S Lehmann R Assmus B Dimmeler S Zeiher AM Transcoronary concentration gradients of circulating microRNAs. Circulation (2011) 124(18):1936–44. 10.1161/circulationaha.111.037572 21969012

[118] Zhou Y Zhou G Tian C Jiang W Jin L Zhang C Exosome-mediated small RNA delivery for gene therapy. Wiley Interdiscip Rev RNA (2016) 7(6):758–71. 10.1002/wrna.1363 27196002

[119] Janssen HL Reesink HW Lawitz EJ Zeuzem S Rodriguez-Torres M Patel K Treatment of HCV infection by targeting microRNA. N Engl J Med (2013) 368(18):1685–94. 10.1056/NEJMoa1209026 23534542

[120] Rupaimoole R Slack FJ. MicroRNA therapeutics: towards a new era for the management of cancer and other diseases. Nat Rev Drug Discov (2017) 16(3):203–22. 10.1038/nrd.2016.246 28209991

[121] Alvarez-Erviti L Seow Y Yin H Betts C Lakhal S Wood MJ. Delivery of siRNA to the mouse brain by systemic injection of targeted exosomes. Nat Biotechnol (2011) 29(4):341–5. 10.1038/nbt.1807 21423189

[122] Bonauer A Carmona G Iwasaki M Mione M Koyanagi M Fischer A MicroRNA-92a controls angiogenesis and functional recovery of ischemic tissues in mice. Science (2009) 324(5935):1710–3. 10.1126/science.1174381 19460962

[123] Hullinger TG Montgomery RL Seto AG Dickinson BA Semus HM Lynch JM Inhibition of miR-15 protects against cardiac ischemic injury. Circ Res (2012) 110(1):71–81. 10.1161/circresaha.111.244442 22052914 PMC3354618

[124] Hinkel R Penzkofer D Zuhlke S Fischer A Husada W Xu QF Inhibition of microRNA-92a protects against ischemia/reperfusion injury in a large-animal model. Circulation (2013) 128(10):1066–75. 10.1161/circulationaha.113.001904 23897866

[125] Abdul-Ghani M Del Prato S Chilton R DeFronzo RA. SGLT2 inhibitors and cardiovascular risk: lessons learned from the EMPA-REG OUTCOME study. Diabetes Care (2016) 39(5):717–25. 10.2337/dc16-0041 27208375 PMC4839176

